# Multivariate Methods for Genetic Variants Selection and Risk Prediction in Cardiovascular Diseases

**DOI:** 10.3389/fcvm.2016.00017

**Published:** 2016-06-08

**Authors:** Alberto Malovini, Riccardo Bellazzi, Carlo Napolitano, Guia Guffanti

**Affiliations:** ^1^Laboratory of Informatics and Systems Engineering for Clinical Research, IRCCS Fondazione Salvatore Maugeri, Pavia, Italy; ^2^Department of Electrical, Computer and Biomedical Engineering, University of Pavia, Pavia, Italy; ^3^Molecular Cardiology Laboratories, IRCCS Fondazione Salvatore Maugeri, Pavia, Italy; ^4^Department of Psychiatry, McLean Hospital, Harvard Medical School, Belmont, MA, USA

**Keywords:** SNPs, multivariate methods, risk scores, risk stratification, cardiovascular diseases

## Abstract

Over the last decade, high-throughput genotyping and sequencing technologies have contributed to major advancements in genetics research, as these technologies now facilitate affordable mapping of the entire genome for large sets of individuals. Given this, genome-wide association studies are proving to be powerful tools in identifying genetic variants that have the capacity to modify the probability of developing a disease or trait of interest. However, when the study’s goal is to evaluate the effect of the presence of genetic variants mapping to specific chromosomes regions on a specific phenotype, the candidate loci approach is still preferred. Regardless of which approach is taken, such a large data set calls for the establishment and development of appropriate analytical methods in order to translate such knowledge into biological or clinical findings. Standard univariate tests often fail to identify informative genetic variants, especially when dealing with complex traits, which are more likely to result from a combination of rare and common variants and non-genetic determinants. These limitations can partially be overcome by multivariate methods, which allow for the identification of informative combinations of genetic variants and non-genetic features. Furthermore, such methods can help to generate additive genetic scores and risk stratification algorithms that, once extensively validated in independent cohorts, could serve as useful tools to assist clinicians in decision-making. This review aims to provide readers with an overview of the main multivariate methods for genetic data analysis that could be applied to the analysis of cardiovascular traits.

## Introduction

The interaction of several genetic and environmental factors modulates the clinical expression of common cardiovascular diseases (CVDs), such as coronary artery disease (CAD), cerebrovascular disease, peripheral arterial disease, and stroke. Poor diet, physical inactivity, smoking, and harmful use of alcohol have all been established as key risk factors that can affect the clinical expression of many CVDs (1). While predisposition to CVD as indicated by the presence of family history suggests that genetic factors play a role in the expression of the trait, the characteristics of inheritance often do not follow Mendelian patterns. For multifactorial diseases, this atypical pattern of inheritance impairs the elucidation of the genetic underpinnings. Indeed, multiple genetic factors with variable effects and effect size have to be identified to account for such a complex “polygenic” inheritance. On the other hand, the variable expressivity commonly found in monogenic cardiac diseases, even among subjects with the same genetic defect, represents a major limitation for the definition of genotype-based risk stratification algorithms (2).

Over the last decade, genome-wide association studies (GWASs) successfully identified more than 1,100 associations of genetic markers with cardiovascular traits, such as stroke, CAD, peripheral arterial disease, variability of the human electrocardiogram, and monogenic cardiac diseases (3). Although providing strong evidence of statistical association with these traits (*p*-value <1 × 10^−8^), single genetic variants identified by GWASs only explain a small proportion of the disease risk or phenotype variability (4–6). As an example, the recently identified CAD-associated variants reviewed in Ref. (4) induce each an average increase in terms of disease risk of ~18% [odds ratio (OR) = 1.18] (5). Further refining in genetic risk prediction and resuming multi-markers information in CVD will require alternative analytical strategies.

In the following sections, this review will address the main multivariate approaches to perform genetic variants selection from GWAS or candidate region studies, how the deriving findings could be modeled to define specific risk profiles and risk stratification algorithms and how to evaluate the prediction accuracy of the defined models.

## Identification of Informative Genetic Variants

Identifying informative genetic markers among millions of candidates generated by microarrays or next generation sequencing (NGS) platforms has historically been a process of ranking variants according to their level of statistical association with a specific trait. This is first estimated by one-SNP-at-a-time testing approaches, and then a subset of these associated variants is selected based on a defined significance threshold (7). More recently, methods have emerged that are better suited for large cohorts of individuals deeply characterized by phenotypic measurements. Multivariate machine learning methods can be applied to identify informative subsets of genetic variants and non-genetic factors that jointly contribute to the overall phenotype expression (8). Annotating the identified markers could then be performed by accessing resources providing information on genomic variants previously associated with a trait of interest (3, 9, 10) and functional annotation tools (11–15). Once validated on independent cohorts of individuals, functional studies will allow researchers to translate evidence of statistical association and informative predictive models into biologically relevant findings (16).

### Multivariate Methods for Common Genetic Variants Selection

Multivariate approaches of feature selection allow researchers to identify a subset or a combination of informative common genetic variants and non-genetic covariates that underlies the risk of developing a trait (17). These approaches offer a method that can overcome the limitations of the one-variant-at-a-time testing strategy characterizing univariate tests, which are incapable of capturing the multifactorial characteristics of many cardiovascular traits (e.g., additive effects of multiple variants, interactions between genetic and non-genetic factors) (18). In general, these approaches select informative variables not based on the strength of their statistical association with the trait, but rather on the basis of their capability to correctly predict the trait value in independent data.

A distinction has then to be made between multivariate methods for the analysis of binary traits (i.e., when the dependent variable indicates the presence or absence of a specific condition) and methods for quantitative traits analysis (i.e., when the dependent variable is characterized by a continuous distribution).

#### Binary Traits Analysis

The analysis of binary traits offers several alternatives that draw from both frequentist and Bayesian methods (Table 1). In order to identify informative sets of genetic and non-genetic variables expected to jointly affect a disease phenotype, stepwise logistic regression is one of the most consolidated approaches. The first step of this approach consists in testing simultaneously an initial set of SNPs in a logistic regression model as predictors of disease status which is represented by the binary-dependent variable. Then, different models are subsequently compared with the initial model to estimate whether a different set of predictors improved the fit, which is measured by goodness of fit metrics such as deviance or log-likelihood (19). Identifying the optimal model can be performed by a forward search strategy (the selection starts with the intercept of the regression, and then sequentially adds into the model the predictor that most improves the fit), a backward search strategy (it starts by including all variables, and sequentially deletes the predictor that has the lowest impact on the fit), or a combination of both (19). However, it is important to consider that this approach may prove computationally intensive when large sets of variables need to be analyzed, making the task of feature selection difficult.

**Table 1:** **Summary of the main multivariate methods for common variants analysis**.

Phenotype	Method	Main software packages	Analysis of entire GWAS datasets	Advantages	Disadvantages
**Binary traits**					
	Stepwise logistic regression (19)	Orange (20), WEKA (21), stats^a^, MASS^a^	Limited to candidate variants	Results can be easily interpreted	Results could be negatively influenced by collinearity; computationally intensive; R implementations^a^ require advanced computer skills
	LASSO (22)	Orange (20), PLINK (23), HyperLASSO (24), glmnet^a^, lars^a^, penalized^a^, ldlasso^a^, scikit-learn^b^	Yes (HyperLASSO), otherwise the analysis is limited to candidate variants	Fast computation; internal CV to learn the optimal λ parameter	Does not necessarily yield good results in presence of high collinearity and when the number of variants exceeds the number of examples; R^a^, Python^b^, and PLINK implementations require advanced computer skills
	Elastic net (25)	elasticnet^a^, glmnet^a^, scikit-learn^b^	Limited to candidate variants	Combines strengths of LASSO and Ridge regression (26), overcoming issues due to collinearity, and unbalanced variants/samples ratio	Requires advanced computer skills
	BOSS (27)	BOSS	Limited to candidate variants	Works properly also when the number of features exceeds the number of samples	Computationally intensive; requires advanced computer skills
	BoNB (28)	BoNB	Yes	Fast computation; robust to LD between variants	Requires advanced computer skills
	Classification trees (29)	Orange (20), WEKA (21), rpart^a^, tree^a^, scikit-learn^b^	Limited to candidate variants	Fast computation; easy to interpret	May not perform well in the presence of complex interactions, overfitting may lead to instability; R^a^ and Python^b^ implementations require advanced computer skills
	Random forest (30)	Orange (20), WEKA (21), randomForest^a^, randomForestSRC^a^, scikit-learn^b^, RFF (31)	Yes (RFF) otherwise the analysis is limited to candidate variants	Robust to noise; fast computation	Results are difficult to interpret; R^a^, Python^b^ and RFF implementations require advanced computer skills
	ABACUS (32)	ABACUS^a^	Candidate regions mapping to specific pathways	Able to simultaneously consider common and rare variants and different directions of genotype effect	Requires advanced computer skills
**Time to event**					
	Stepwise Cox proportional hazard model	Survival^a^, MASS^a^	Limited to candidate variants	Results can be easily interpreted	Results could be negatively influenced by collinearity; computationally intensive; requires advanced computer skills
	LASSO (22)	glmnet^a^, penalized^a^ coxnet^a^	Limited to candidate variants	Fast computation; internal CV to learn the optimal λ parameter	Does not necessarily yield good results in presence of high collinearity and when the number of variants exceeds the number of examples; requires advanced computer skills
	Elastic net (25)	coxnet^a^	Limited to candidate variants	Combines strengths of LASSO and Ridge regression (26), overcoming issues due to collinearity, and unbalanced variants/samples ratio	Requires advanced computer skills
	Classification (survival) trees (29)	rpart^a^	Limited to candidate variants	Fast computation; easy to interpret	May not perform well in the presence of complex interactions, overfitting may lead to instability; requires advanced computer skills
	Random forest (30)	randomForestSRC^a^	Limited to candidate variants	Robust to noise; fast computation	Results are difficult to interpret; requires advanced computer skills
**Quantitative traits**					
	Stepwise linear regression	stats^a^, MASS^a^	Limited to candidate variants	Results can be easily interpreted	Results could be negatively influenced by collinearity; computationally intensive; requires advanced computer skills
	LASSO (22)	Orange (20), PLINK (23), HyperLASSO (24), glmnet^a^, lars^a^, penalized^a^, ldlasso^a^, scikit-learn^b^	Yes (HyperLASSO), otherwise the analysis is limited to candidate variants	Fast computation; internal CV to learn the optimal λ parameter	Does not necessarily yield good results in presence of high collinearity and when the number of variants exceeds the number of examples; R^a^, Python^b^, and PLINK implementations require advanced computer skills
	Elastic net (25)	Elasticnet^a^, glmnet^a^, scikit-learn^b^	Limited to candidate variants	Combines strengths of LASSO and Ridge regression (26), overcoming issues due to collinearity, and unbalanced variants/samples ratio	Requires advanced computer skills
	GUESS (33)	GUESS/R2GUESS^a^	Yes	Fast parallel computation	Requires advanced computer skills
	Regression trees (29)	Orange (20), rpart^a^, tree^a^, scikit-learn^b^	Limited to candidate variants	Fast computation; easy to interpret	May not perform well in the presence of complex interactions, overfitting may lead to instability; R^a^ and Python^b^ implementations require advanced computer skills
	Random forest (30)	Orange (20), randomForest^a^, randomForestSRC^a^, scikit-learn^b^, RFF (31)	Yes (RFF) otherwise the analysis is limited to candidate variants	Robust to noise; fast computation	Results are difficult to interpret; R^a^, Python^b^, and RFF implementations require advanced computer skills

*Phenotype, dependent variable’s distribution; method, algorithm or method; main software packages, main softwares, packages, or functions implementing the described method; analysis of entire GWAS datasets, indicates whether the method can be applied to whole GWAS data; advantages, advantages of the method; disadvantages, disadvantages of the method*.

*^a^R package*.

*^b^Python package*.

The Least Absolute Shrinkage and Selection Operator (LASSO) (22) is a shrinkage method that represents a sound alternative to stepwise regression for the identification of informative genetic variants. The LASSO approach silences non-informative variables by setting their regression coefficient to 0 through a penalty parameter called lambda (λ). The optimal value to be assigned to λ can be learned by a resampling strategy performed on the data: the value guaranteeing the lowest average classification error on the test sets will be applied to the regression model. Vaarhorst and colleagues (34) used LASSO to identify predictors of coronary heart disease (CHD), starting from a set of candidate variants, whereas Hughes and colleagues (35) applied the algorithm to the identification of genetic variants to define a risk score for coronary risk prediction. The elastic net (25) is an extension of the LASSO that is robust to extreme correlations among predictors, which also provides a more efficient, effective system for handling the analysis of unbalanced datasets.

Bayesian methods, such as the binary outcome stochastic search (BOSS) (27) and bags of naive Bayes (BoNB) (28) algorithms, also provide alternative approaches. BOSS is a feature selection approach deriving from the method described in Ref. (36) based on a latent variable model that links the observed outcome to the underlying genetic variants mapping to candidate regions of interest. A Markov Chain Monte Carlo approach is used for model search and to evaluate the posterior probability of each predictor in determining the latent variable profile (27). A latent variable profile is defined as a stochastic vector of same size of the number of SNPs; the vector may assume 0/1 values, thus expressing the fact that a marker is considered (value equal to 1) or not (value equal to 0) as a predictor of the outcome. The model estimates the posterior probability of such latent variable; as a consequence, the most likely latent variable will determine the set of SNPs with the highest risk prediction potential for developing a disease. BoNB (28) is an algorithm for genetic biomarkers selection from the simultaneous analysis of genome-wide SNP data based on the naive Bayes (NB) (37) classification framework. The predictive value (marginal utility) of each genetic variant is assessed by a resampling strategy. By randomly shuffling the genotypes of an informative variant, an overall decrease in terms of classification accuracy will be observed, and if an uninformative variant is permuted, no substantial loss will be observed. This strategy, coupled with appropriate statistical tests, allows BoNB to identify informative sets of SNPs. These methods have been tested on real datasets on type 1 (28, 38) and type 2 diabetes (27), respectively.

Classification and regression trees (RTs) methods (29) fall under the category of decision tree learning. In these tree structures, leaves represent the predicted phenotypic outcome, whereas nodes and branches represent the set of genetic variants and clinical covariates that predict the phenotypic outcome. These methods recursively partition data into subsets according to the variables’ values: each partition corresponds with a “split” based on the set of variables being considered, defining a tree-like structure (19). Classification trees (CTs) are designed to analyze categorical traits and facilitate the identification of informative interactions between variables and stratifications in the data starting from a limited numbers of predictors.

Random forests (RFs) (30) are based on CTs, as they aggregate a large collection of de-correlated trees, and then average them (19). RFs generate a multivariate ranking of the analyzed variables according to their predictive importance with respect to the outcome. Even more, they can be easily applied to analyze unbalanced datasets, and they are able to account for correlation and informative interactions among features. Such characteristics make this approach particularly appealing for high-dimensional genomic data analysis (39). RFs have been applied to identify genetic variants influencing coronary artery calcification in hypertensive subjects (40), bicuspid aortic valve condition (41), and high-density lipoprotein (HDL) cholesterol level (42). Maenner and colleagues (43) applied RFs to identify SNPs involved in gene-by-smoking interactions related to the early-onset of CHD using the Framingham Heart Study data.

ABACUS is an Algorithm based on a BivAriate CUmulative Statistic, which allows identifying combinations of common and rare genetic variants associated with a disease by focusing on predefined SNPs-sets (e.g., belonging to specific pathways) (32). ABACUS calculates a statistic for each pair of SNPs within each SNPs set and generates an aggregated score measuring the cumulative evidence of association of the SNPs annotated in the SNP set. This method has been tested on GWAS on type 1 and type 2 diabetes (32).

Specific implementations of LASSO, elastic net, CTs, RFs, and stepwise Cox proportional hazard regression (44) have been also proposed for the identification of SNPs associated with time to event outcomes (Table 1).

#### Quantitative Traits Analysis

Many of the feature selection methods for binary traits derive from algorithms originally established for quantitative traits analyses (Table 1). Linear regression (45) coupled with stepwise feature selection is probably one of the most commonly applied approaches when dealing with the task of identifying informative predictors with respect to continuous traits starting from a limited set of variables.

The LASSO and the elastic net shrinkage algorithms for regression problems work similarly for classification. Warren and colleagues (46) used LASSO and HyperLASSO (24) to predict low-density lipoprotein (LDL) and HDL cholesterol, two lipid traits of clinical relevance. Bottolo and colleagues (33) published the results from the validation and implementation of a method called Graphical Unit Evolutionary Stochastic Search (GUESS), a Bayesian variable selection approach able to analyze single and multiple responses, searching for the best combinations of SNPs to predict the traits. The authors applied the method to study genetic regulation of lipid metabolism in the Gutenberg Health Study (GHS), confirming the association of previously identified loci for blood lipid phenotypes.

Though largely similar to CTs, RTs differ from CTs in that the dependent variable is continuous, and a regression model is fitted to each node to perform the task of prediction. Additionally, RFs for regression problems are also widely employed and implemented in specific analytical packages.

## Multivariate Models for Decision Support

Demographic, clinical, and genetic risk factors identified by the previously described methods or selected based on prior knowledge can be combined in order to define specific predictive models, which could assist clinicians during the decision make steps of the clinical practice (47–49). Such models can be defined by making use of the above mentioned methods. For example, multilocus genetic risk profiles can be defined by weighting genetic variants by the corresponding regression coefficients (50, 51). Similarly, tree-based approaches or regression methods can be applied to define risk stratification algorithms combining genetic and non-genetic information (49, 51).

### Multilocus Genetic Risk Profiles

The theory of multifactorial, polygenic liability relies on the combined effect of multiple common genetic variants, each explaining a small amount of phenotypic variance and possibly interacting with environmental factors, all contributing to the overall risk (52, 53). Polygenic risk score (PRS) approaches were introduced to examine the load of genetic risk associated with a given disease by simultaneously testing a broad set of common variants (54). Essentially, the PRS approach capitalizes on the identification of genetic risk variants derived from large, mega-, or meta-analyses for specific disorders and generates an index of genetic vulnerability associated with the disease (54). Affected subjects present higher values of the PRS than not affected subjects. The advantage of polygenic modeling is that the genetic vulnerability is represented by a larger set of gene-mapping variants contributing to the risk of the disease, rather than a single genetic variant. There are several different ways to implement polygenic modeling approaches (55). All methods rely on selecting variants on a training set using univariate or multivariate approaches or focusing on candidate loci identified by previous studies. The risk alleles of the identified sets of genetic variants are then used to generate a PRS either by summing the number of risk alleles (“un-weighted” approach) or by weighting the number of risk alleles by the effect size of the association deriving from regression models (“weighted” approach) (50). Either way, the PRS is tested for association in a replication sample *via* traditional regression-based statistics and standard metrics are used to estimate its predictive power (56).

Polygenic risk score usually explain 1–5% of the variation in complex traits, which is already an improvement compared with GWAS single genetic variants, which typically yield relatively small increment of risk with ORs <1.5-fold, with the exception of traits such as height, for which a GWAS identified a SNP explaining almost 5% of the phenotypic variance (53, 57). PRS have been applied to several CVD studies and are found to be a significant predictor of CAD (58, 59), incident cardiovascular (60), CHD (61), atrial fibrillation, and stroke (62). Furthermore, Pfeufer and colleagues (63) assessed the cumulative effect of SNPs modulating the QT interval in the general population. For a more comprehensive review of PRS findings in CVD, we encourage readers to consider the report by Abraham and Inouye (51).

### Risk Stratification Algorithms

Risk stratification algorithms are designed to be intuitive tools that can assist clinicians in identifying patients at high risk of adverse events, thus informing decision-making by following a defined set of logical steps (64–66). These algorithms can be derived by the integration of genetic information (e.g., single SNPs, mutations on causative loci, PRSs) with known clinical and behavioral risk factors by appropriate multivariate methods. When defined by regression methods, they can be interrogated by nomograms, graphical tools that allow interpreting the risk of developing a certain trait based on an individual’s characteristics (67).

**Figure 1:**
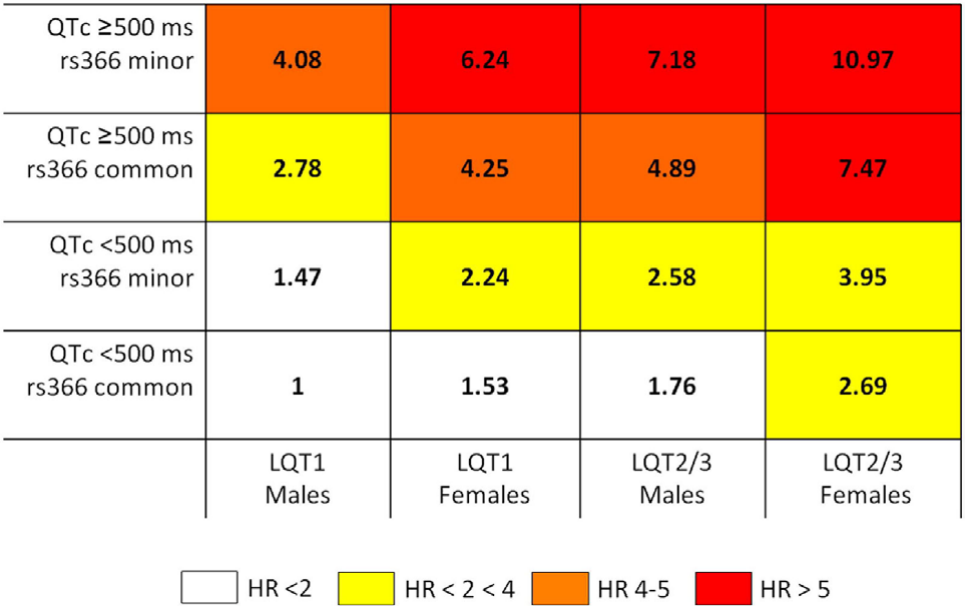
**rs10494366 common variant on *NOS1AP* modulates risk of events in LQTS (48)**. The schema reports the combined hazard ratios (HRs) from Cox regression by risk categories. The risk stratification schema includes the common variant rs10494366 on *NOS1AP* gene and known risk predictors in LQTS, represented by: QTc ≥ 500 ms, gender, and LQTS subgroup. Each box shows the combined HR for patients sharing clinical and genetic characteristics. The reference category (HR = 1) is represented by individuals LQT1, males, QT < 500 ms and homozygote for the common allele of *NOS1AP* rs10494366. Reprinted from the manuscript by Tomás and colleagues (48) with permission from Elsevier.

Priori et al. (47) proposed a risk stratification algorithm to identify long QT syndrome (LQTS) patients at high risk of adverse cardiac events (defined as occurrence of syncope, cardiac arrest, or sudden death before the age of 40 years and in absence of therapies). LQTS is a genetic disorder caused by mutations that affect ion-channel encoding genes or other genes that indirectly modulate the function of ion channels. The algorithm was based on the combination of information about the presence of genetic variants on one of the three main LQTS genes (*KCNQ1*, *KCNH2*, and *SCN5A* defining LQT1, LQT2, and LQT3), gender, and QT interval duration (≥500 or <500 ms), which are known independent risk predictors in LQTS. Three risk groups were identified based on the observed probability of an adverse cardiac event: low risk (probability <30%), intermediate risk (30–49%), and high risk (≥50%). Based on the published risk stratification algorithms for LQT1, LQT2, and LQT3 patients (47, 68), Tomás and colleagues (48) investigated whether common variants on *NOS1AP* locus can add additional insights for risk stratification in this group of patients. The authors demonstrated that the presence of the *NOS1AP* rs10494366 variant improved event risk stratification for previously identified LQT1, LQT2, and LQT3 patients. The presence of the GG or GT genotype of *NOS1AP* rs10494366 increased the risk of cardiac events compared with homozygotes for the T allele in all the subgroups of LQTS patients defined by different combinations of gender and genetic locus (Figure 1).

Talmud et al. (69) evaluated whether the inclusion of information regarding the genotype of rs10757274 on 9p21.3 locus to the risk factors defining the Framingham risk score (FRS) allowed increasing the accuracy in identifying patients at risk of CHD in a prospective study. Results showed that, although rs10757274 did not add substantially to the usefulness of the FRS for predicting future events, it did improve reclassification of CHD risk, and thus may have clinical utility.

Ripatti et al. (58) tested 13 SNPs – associated with myocardial infarction or CAD by previous GWASs – in a case–control design including 3,829 CHD cases and 48,897 control participants and a prospective cohort design including 30,725 individuals free of CVD. In prospective cohort analyses, the weighted PRS defined using the set of selected SNPs was significantly associated with a first CHD event. Furthermore, when compared with the bottom quintile of the PRS distribution, individuals in the top quintile shared a 1.66-fold increased covariates-adjusted risk of CHD. When focusing on its risk prediction capability, the PRS did not improve the C index over clinical risk factors but increased slightly the integrated discrimination index (*p*-value <0.001). Similar results were obtained from the case–control analyses.

## Model Assessment Strategies

Once multivariate sets of SNPs, PRSs or risk stratification algorithms are defined on an initial cohort (training set), their accuracy in predicting the condition of new examples must be assessed on independent populations (test set). In the absence of independent cohorts, it is possible to rely on resampling strategies like K-Fold Cross Validation (K-Fold CV) (19), holdout (70), and bootstrap (71). Several metrics are available to evaluate and compare the discriminative power of predictive models on the test set, based on the trait’s distribution (72, 73).

## Conclusion

The goal of this review is to provide readers with an overview about the main multivariate methods that can be applied to the identification of informative genetic variants and to the definition of risk prediction tools in the context of CVDs. It is important to note that some methods described have been applied to intermediate phenotypes that could be considered precursors to their manifestation as cardiovascular traits, but these methods have not yet been applied to the analysis of cardiovascular traits. Their application to large CVD cohorts could lead to interesting findings.

Multivariate methods allow the identification of complex additive effects due to the presence of multiple genetic variants on specific loci or complex interactions among genetic and non-genetic risk factors able to modulate the probability of developing a specific disease or its severity.

Still, the task of identifying informative combinations of genetic variants by multivariate search strategies can be extremely computationally intensive due to the high number of models to be explored and, in many cases, to the impossibility of parallelizing the analyses. Missing values represent a common limitation to these approaches, although it could be partially solved by resorting to multivariate imputation methods. Furthermore, large sets of samples thoroughly characterized in terms of phenotype characteristics are needed in order to avoid overfitting issues and to increase the probability of defining models whose predictive performances can be confirmed in independent cohorts.

## Author Contributions

AM and GG conceived the study and drafted the manuscript. RB and CN conceived the study and revised the manuscript critically for important intellectual content. All authors approved the final version of the manuscript.

## Conflict of Interest Statement

The authors declare that the research was conducted in the absence of any commercial or financial relationships that could be construed as a potential conflict of interest.
